# Parental factors associated with walking to school and participation in organised activities at age 5: Analysis of the Millennium Cohort Study

**DOI:** 10.1186/1471-2458-11-14

**Published:** 2011-01-06

**Authors:** Sinead Brophy, Roxanne Cooksey, Ronan A Lyons, Non E Thomas, Sarah E Rodgers, Michael B Gravenor

**Affiliations:** 1 Centre for Health Information, Research and Evaluation, School of Medicine, Swansea University, Singleton Park, Swansea, Wales, SA2 8PP, UK; 2 Centre for Child Health, School of Human Sciences, Swansea University, Singleton Park, Swansea, Wales, SA2 8PP, UK

## Abstract

**Background:**

Physical activity is associated with better health. Two sources of activity for children are walking to school and taking part in organised sports and activities. This study uses a large national cohort to examine factors associated with participation in these activities.

**Methods:**

The Millennium Cohort study contains 5 year follow-up of 17,561 singleton children recruited between 2000-2002 in the UK. All participants were interviewed in their own homes at 9 months, 3 years and 5 years follow-up and all measures were self reports. Logistic regression and likelihood ratio tests were used.

**Results:**

Children are less likely to walk to school as income and parental education increase [Adjusted odds: 0.7 (95%CI: 0.6-0.8) for higher income/education compared to low income/no qualifications]. However, if the parent plays with the child in high income families the child is more likely to walk to school [Adjusted odds: 1.67 (95%CI: 1.3-2.1)]. Children taking part in organised activities are from higher income, higher education families, with a car, in a "good" area with non-working mothers. However, in low socio-economic families where the parent plays with the child the child is more likely to take part in organised activities [Adjusted odds: 2.0 (95% CI: 1.5-2.7)].

**Conclusions:**

Income is an important determinant of the type of activity available to children. Families that report good health behaviours (non-smoking, low TV viewing) and play with their children show higher levels of physical activity. Thus, parenting practice appears to have a strong impact on their child's physical activity.

## Background

Levels of physical activity in children are declining [1-4] while childhood obesity is receiving increasing attention [5-7] and reported to have doubled over the last two decades in the UK [8]. There is overwhelming evidence that sources of physical activity, such as walking to school, which can form a routine activity built into the normal day, are dramatically declining [9-12]. Participation in activity is associated with positive self beliefs and better long term health including improved cardiovascular and musculoskeletal fitness and weight reduction [13-15]. However, physical activity and fitness levels are declining among young people with time [2,12]. This change in levels of activity can have long term implications as physical activity levels are thought to be developed from a young age and behaviours established when young persist into adulthood [16]. Therefore, it is crucial to target and increase physical activity levels of children in order for health benefits to be gained over a lifetime. Changes in society have led to children having less independent play outside, more TV viewing and motorised travel. Additionally, concerns regarding safety and injury for the child mean that more children spend their time supervised [17].

Most research to date has focused on older children (aged 9+) and adolescents [18-21] and have been conducted in urban areas in the U.S. and Australia, which are both countries with urban design not commonly found in other regions such as Europe. In addition, many studies have focused on predominantly white children. Thus, there is a need to examine different regions, younger ages and to include ethnic minority children, as obesity rates vary with ethnicity [22-25]. This study uses existing data from the Millennium cohort study to examine factors associated with activity in children in the UK aged 5 (first year in school) who are very strongly reliant on parents to walk them to school and take them to supervised activities, to the park and to play with them. The aim is to inform the development of interventions to improve physical activity levels in younger children with a focus on ethnic minority children.

## Methods

### Millennium Cohort Study

This study used data collected by the Millennium Cohort Study (MCS) [19] which is described in detail in online technical reports [19]. The MCS follows the lives of a sample of 18,552 babies born between 1 September 2000 and 31 August 2001 in England and Wales, and between 22 November 2000 and 11 January 2002 in Scotland and Northern Ireland. Families with children who were living in the UK at age 9 months and eligible to receive Child Benefit at that age were invited to participate (72% response rate)[26]. Subsequent interviews were carried out at the second contact (78% response rate) and third contacts (79.2% response rate) when children were approximately 3 and 5 years of age, respectively [27]. A stratified cluster sampling framework was employed to adequately represent families from disadvantaged areas and ethnic minority groups [26]. Parents were interviewed in the home and over 99% of the main respondents interviewed were the natural mothers [28]. Data from each of the three surveys were obtained from the UK Data Archive, University of Essex.

Sub-group of children examined: Only the singleton children (of the 18,552 children recruited at the first sweep of the MCS) were included within this study. The ethnic group of the child was recorded at the first contact using the 11 Category Census Classification (UK) and only children designated to either White, Asian or African ethnic group were included in the study (n = 17,561). The group defined as Asian included families recorded as; Indian, Pakistani, Bangladeshi and Other Asian, the group defined as African included families recorded as Black Caribbean, Black African and Other Black. The child's BMI (weight (kg))/(height in meters)2) was measured by trained interviewers and classified using the International Obesity Task Force (IOTF) cut-offs for BMI which are age and sex specific [24].

### Outcomes and potential risk factors

Factors associated with walking to school and/or structured activities were divided into categories of: demographic variables, socio-economic variables, housing and area, parental factors and factors associated with the child (Table 1). All the variables were collected from self-report by the respondent (usually the mother) and no independent measurements of activity (e.g. accelerometer readings or physical activity questionnaires) were performed. Details of data collection are published elsewhere [26,27]. Only those who participated at age 5 were analysed. Outcomes were based on questionnaire reports completed by the primary carer, about the child walking to school, and of the number of days per week the child takes part in organised sports or activities. Factors examined are given in Tables 2 and 3.

**Table 1:** Variables examined

Variable	Sweep of data collection	Units of analysis
*Demographic variables*		
Sex	First	Male or female
Ethnic Group	First	White, Asian (includes Indian, Pakistani, Bangladeshi and other Asian), African (includes Black Caribbean, Black African, Other Black)
Number in household	First	Number reported
*Socio-economic variables*		
Family Income	First	Per annum: <£10,400, £10,400 - £20,800 >£20,800
Highest academic qualification	First	None, GCSE/O'level, A'level, University degree or higher
Access to car or van	Second	Yes or no
In work	Third	Yes or no
*Housing/Area*		
Good area to raise children	Third	Poor, good or excellent
Area/setting	First and second	Urban, town or village
Type of accommodation	Second and third	House/bungalow or Flat/maisonette
Access to garden	First, second and third	Yes or no
Perceived safety of area	Third	Safe, moderate, unsafe
*Parental factors*		
Age of respondent	Third	Age reported
Respondent general health	Third	Excellent, very good, good, fair or poor
Anyone smoke near child	Third	Yes or no
*Child factors*		
BMI of child	Third	BMI score reported
Parental assessment child behaviour (SDQ)	Third	SDQ score reported
Bracken score	Second	Bracken score reported

Exercise outside school at a club or class	Third	1 = Once a week, 0 = Less than once a week
Walk or cycle to school	Third	Yes or no
Sport with parent	Third	1 = Once a week, 0 = Less than once a week
Plays physically active games with parent	Third	1 = Once a week, 0 = Less than once a week
Go to playground with parent	Third	1 = Once a week, 0 = Less than once a week
Hours television watched per day	Third	Less that 3 hours or more than 3 hours

**Table 2:** Crude odds of factors associated with walking to school

Variable	% walking to school in the exposed group	% walking to school in the unexposed group	Odds ratio * (exposed/unexposed)
*Demographics*			
Male/Female	Male: 50.7% (3538/6976)	Female: 50.5% (3369/6671)	-
Ethnic group	African/Asian: 60.5% (1071/1770)	White: 49.1% (4356/8729)	1.59 (95%CI: 1.4-1.75)
Number in household	More than 4: 51.8% (2551/4918)	Four or less: 49.9% (4356/8729)	1.04 (95%CI: 1.02-1.07)

*Socio-economics*			
Family income	Low 62% (1930/3113)	Medium: 53% (2208/4157) High: 41% (3089/5283)	1.5 (95%CI: 0.1.45-1.59)
Academic achievement	None: 64% (1508/2339) O'level: 52% (3121/6059)	A'Level: 47% (634/1336) Uni :40% (1431/3542)	1.6 (95%CI: 1.56-1.79)
Access to car	Have a car: 45.9% (4844/10559)	No access to car: 78.5% (1339/1706)	0.23 (95%CI: 0.21- 0.26)
Primary carer in work	In work: 44% (3430/7815)	Not in work: 60% (3477/5831)	0.53 (95% CI: 0.50-0.57)

*Area*			
Good area to raise children	Excellent:47.2% (4592/9717)	Moderate: 58% (1696/2939) Poor/very poor: 62.9% (607/964)	0.62 (95% CI: 0.58-0.67)
Area/Setting	Urban: 54.5% (4868/8926)	Town: 48.8% (421/862) Village: 37.9% (266/702)	1.5 (95%CI: 1.37-1.7)
Perceived safety of area	Very safe: 49% (5798/11779)	Moderate-not safe: 59% (1095/1846)	0.66 (95%CI: 0.6-0.7)

*Parents*			
Age of primary carer	More than 35 yrs: 45.4% (2563/5651)	Less than or equal to 35 yrs: 54.3% (4344/7996)	0.7 (95%CI: 0.65-0.74)
Health of primary carer	Good: 50.3% (6659/13,231)	Poor: 59.1 (240/406)	0.7 (0.57-0.86)
BMI of primary carer	More than 25: 49.9% (2355/4717)	Less than or equal to 25: 49.1% (3499/7114)	-
Smokes near child	Smokes: 59.7% (1167/1954)	Does not smoke: 49% (787/6736)	1.53 (95% CI: 1.4-1.7)

*Child*			
BMI of child (overweight/obese according to Obesity Taskforce definition)	Overweight/obese: 50.3% (1466/2914)	Not overweight/obese: 50.7 (5345/10548)	-
Bracken School Readiness Score	Less than 3: 56.3% (2273/4037)	More or equal to 3: 48.2 (4634/9610)	1.38 (95%CI: 1.2-1.5)
Strengths and Difficulties Questionnaire total >7	More than 7: 54.4% (3695/6786)	Less or equal to 7: 46.8% (3212/6907)	1.35 (95%CI: 1.3-1.45)
Taking part in organised sports/exercise	Organised sport: 45.5% (3223/6906)	No organised sport: 56.1% (3683/6562)	0.65 (95%CI: 0.61-0.70)
Plays sports (with parent)	Plays sport with parent: 50.0% (4667/9341)	Does not play sport with parent: 52.0% (2239/4305)	0.92 (95%CI: 0.85-0.99)
Playing physically active games (with parent)	Plays games: 50.6% (4145/8190)	Does not play games: 50.58% (2757/5451)	-
Going to playground (with parent)	Goes to play ground: 52.7% (4469/8469)	Does not go to playground: 47.1% (2432/5167)	1.25 (95%CI: 1.17-1.3)
More than 3 hours of TV per day	More than 3 hours: 56% (1145/2042)	Less than 3 hours: 49.6% (5758/11599)	1.29 (1.17-1.4)

* The tables are set out so that the exposed group is always compared to the unexposed group. For example, on table 2 exposures is taken as being of an ethnic minority group - so exposed/unexposed would be the proportion walking in the exposed group (ethnic minority) divided by the proportion walking in the unexposed group (white).

**Table 3:** Crude odds for factors associated with taking part in organised exercise

Variable	% taking part in organised activity in the exposed group	% taking part in organised activity in the unexposed group	Odds ratio (exposed/unexposed)
*Demographics*			
Male	Male: 48.3% (3436/7112)	Female: 55.39% (3750/6770)	0.75 (95%CI:0.7 -0.8)
Ethnic group	Asian/African: 24.1% (434/1797)	White: 55.9% (6752/12085)	0.25 (95%CI: 0.22-0.28)
Number in household	Greater than 4: 44.6% (2234/5010)	Equal or less than 4: 55.8 (4952/8872)	0.64 (95%CI:0.59-0.68)

*Socio-economics*			
Family income	Low 31.9% (1012/3165)	Medium: 45.2% (1915/4234) High: 69.1% (3713/5371)	0.33 (95%CI: 0.3-0.36)
Academic achievement	None: 26.3% (627/2381) O'level: 48.8% (3000/6148)	A Level: 62.5% (853/1364) Uni: 71.7% (2586/3608) A'level: 7.9% (511/6435) Uni: 15.9% (1022/6435)	0.33 (95% CI: 0.31-0.36)
Access to car or van	Access to car: 57.3 (6147/10736)	No access to car: 26.0% (453/1740)	3.8 (95%: 3.4-4.2)
Employment status	Mother Employed: 61% (4831/7933)	Mother unemployed: 40% (2354/5948)	2.4 (2.22-2.56)

*Area*			
'Good' area to raise children	Excellent: 57.4% (5676/9890)	Moderate: 40.5% (1208/2985) Poor/very poor: 29.9% (293/980)	2.2 (2.1-2.4)
Area/Setting	Urban: 47.2% (4269/9031)	Town: 60% (527/876) Village: 66.6% (470/706)	0.59 (95%CI: 0.5-0.68)
Perceived safety of area	Very safe: 53.1% (6939/13081)	Moderate-not safe: 30.8% (247/801)	2.5 (2.2-2.9)
Access to garden	Have access to garden: 53.3% (95%CI: 6746/12653)	No access to garden: 35.7% (435/1220)	2.06 (95%CI: 1.8-2.3)

*Parents*			
Primary carer >35 years of age	Mother added >35: 59.2 (3977/6714)	Mother less than 35 years: 44.8 (3209/7168)	1.8 (95%CI:1.7-1.9)
Health of primary carer	Good: 52.3% (7046/13459)	Fair/poor: 33.1% (140/423)	2.2 (95% CI: 1.8-2.7)
BMI of primary carer >25	BMI > 25: 50.3% (2417/4803)	BMI less than or equal to 25: 55.4 (4009/7232)	0.81 (95%CI: 0.75-0.88)
Smokes near child	Smokes: 32% (637/1993)	Does not smoke: 55.1% (6543/11876)	0.38 (0.34-0.42)

*Child*			
BMI of child (overweight)	Child overweight: 51.6% (1522/2950)	Child not overweight 52.2% (5604/10742)	0.98 (95%CI: 0.9-1.1)
Bracken School Readiness	Score less than 3: 32.5% (497/1531)	Score 3 or more: 54.2 (6689/12351)	0.4 (95%CI: 0.36-0.46)
Strengths and Difficulties Questionnaire total is >7	Score more than 7: 42.5% (2940/6925)	Score 7 or less: 61% (4246/6957)	0.47(95%CI: 0.44-0.5)
Walking to or from school	Walks: 46.7% (3223/6906)	Drives: 57.3% (3861/6740)	0.65 (95%CI: 0.6-0.7)
Plays sports (with parent)	Plays sports: 57.2% (5440/9530)	Does not play sports: 39.9% (1746/4379)	2.0 (95%CI: 0.88-2.2)
Plays physically active games (with parent)	Plays games: 56.4% (4700/8331)	Does not play games: 44.8% (2484/5546)	1.6 (95%CI: 1.5-1.7)
Go to playground	Goes to playground: 53.8% (4642/8626)	Does not go to playground: 48.4% (2537/5246)	1.2 (95%CI: 1.2-1.3)
More than 3 hours of TV per day	Watches more than 3 hours of TV: 40.7% (851/2089)	Watches less than 3 hours of TV: 53.7% (6333/11788)	0.59 (95%CI: 0.53-0.65)

### Statistical analysis

STATA release 8 was used for all analysis. Factors associated with child activity were examined for evidence of confounding or interaction with parental income and length of time in education (as measures of socio-economic status) and ethnic background (White/European, South Asian or African), using Mantel-Haenszel tests followed by regression analysis using likelihood ratio tests. Logistic regressions were performed using all factors associated with activity in an initial unadjusted analysis and likelihood ratio tests were used to build the adjusted model. Interaction terms for variables in the adjusted model were examined using likelihood ratio tests. Goodness of fit was assessed using the Hosmer and Lemeshow statistic [29]. Risk ratios for subgroups were also calculated to facilitate interpretation of findings.

## Results

### Walking to school

Children were less likely to walk to school as income and education levels of the primary carer increased [Odds ratio: 0.7 (0.6-0.8) see Tables 2 and 4]. Walking to school was more likely if the primary carer was not working, the family did not have access to a car, and if the child lived in an urban area (as opposed to a village) [See Table 4]

**Table 4:** Regression analysis. Adjusted odds* for walking to school

Risk factor	Adjusted Odd ratio	95%CI
Education: Primary carer has O'levels (education until age 16, compared to no qualifications)	0.82	0.71-0.94
Education: Primary carer has A'levels (education until age 18, compared to no qualifications)	0.75	0.62-0.90
Education: Primary carer has University qualifications (education to age 20+, compared to no qualifications)	0.69	0.59-0.81
No access to car or van	2.79	2.38-3.28
Primary carer is not working	1.39	1.26-1.52
Living in town (compared to Urban)	0.94	0.80-1.09
Living in village (compared to urban)	0.71	0.60-0.84
Doing organised sports (Low income family)	0.58	0.47-0.71
Doing organised sports (Middle income family).	1.59	1.2-2.0
Doing organised sports (High income family)	1.67	1.3-2.1
Middle income (compared to low income)	0.99	0.8-1.2
High income (compared to low income)	0.73	0.6-0.8
Parent takes child to playground	1.18	1.1-1.3

Adjusted odds means that they are entered into a regression model together and all the variables are therefore adjusted for the other variables in the model.

Factors no longer related to walking to school included: ethnic group, health of parent, smoking near child, Bracken School Readiness Score, Strengths and Difficulties score, watching TV, living in a good area to raise a child, age of parent, playing physically active games, sex ratio, BMI of primary carer.

**Figure 1:**
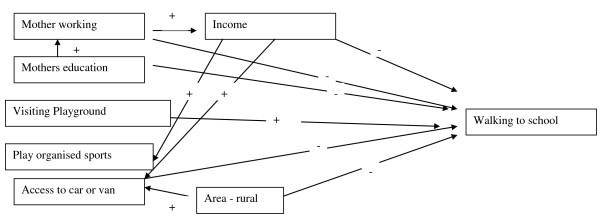
**Diagram of factors associated with walking to school based on findings from the regression analysis**.

However, in physically active high income families (i.e. children who were from higher income families and who took part in organised sports) children were also likely to walk to school [odds ratio 1.7 (95%CI 1.3-2.1) See Table 4]. Thus, children are more likely to walk to school if they are from low income backgrounds, or are active affluent children [See Table 4]. Factors associated with walking to school are given in Figure 1.

### Organised sports and activities

**Figure 2:**
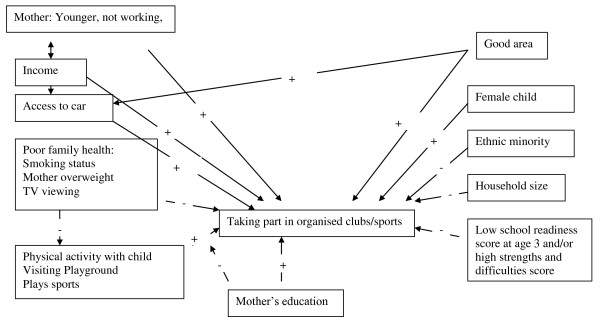
**Diagram of factors associated with taking part in organised sports based on findings from the regression analysis**.

Children from ethnic minority groups, those with no access to a car, from larger families, with a working mother, an overweight mother and in a family with poor health behaviours (smoking, watching >3 hour TV) were less likely to participate in organised exercise [Tables 3 and 5]. Children who were more likely to take part in organised activities were; girls, those with higher school readiness scores, those with younger parents, where the mother has a higher educational achievement level, higher income families and children in excellent areas to raise a child. However, maternal education plays a role. When maternal education is high, parents who play sports with their child are less likely to bring their child to organised clubs. Yet, when the mother has no qualifications, but the family play sports with their child, they are also more likely to bring their child to organised sports and clubs. The proposed model for factors associated with taking part in organised activity is given in Figure 2.

**Table 5:** Regression analysis. Adjusted odds* of taking part in organised clubs or sports

Risk factor	Adjusted Odd ratio	95%CI
Asian or African Child	0.43	0.35-0.53
Number in household (change for each additional member of the household)	0.92	0.88-0.96
Female child	1.36	1.23-1.5
Education: O'level compared to no qualifications	1.9	1.5-2.5
Education: A'level compared to no qualifications	2.5	1.8-3.6
Education: University compared to no qualifications	3.3	2.4-4.5
No access to car or van	0.6	0.5-0.7
Working mother	0.83	0.75-0.92
Middle income compared to low income	1.1	0.96-1.2
Higher income compared to low income	1.7	1.4-1.9
Area (comparison average area with poor area to raise a child)	1.04	0.84-1.3
Area (comparison of 'excellent' area with 'poor' area to raise a child)	1.4	1.12-1.7
Smoking near child	0.57	0.5-0.67
Mothers age (under 35 compared to over age 35)	1.13	1.03-1.25
Mother overweight or obese (compared to normal weight)	0.9	0.82-0.99
School Readiness Score (for each point improvement on score)	1.22	1.1-1.3
Strengths and Difficulties Questionnaire (for each point increase in difficulties)	0.97	0.96-0.98
Parent plays with child (no qualifications)	2.0	1.5-2.7
Parent plays with child (O'level)	0.62	0.45-0.86
Parent plays with child (A'level)	0.65	0.42-0.99
Parent plays with child (Uni)	0.58	0.4-0.8
Plays physically active games	1.11	1.0-1.2
Takes child to playground	1.11	1.0-1.22
Child watches more than 3 hour TV per day	0.85	0.74-0.97

*Adjusted odds means that they are entered into a regression model together and all the variables are therefore adjusted for the other variables in the model.

Factors no longer related to participating in activities included; walking to school, perceived safety of area, health of parent, access to a garden, area/setting (urban, rural, village).

### Goodness of fit

Goodness of fit assessment showed no significant difference between the models and the observed data, confirming a good fit of both model to the data (p = 0.43 for Table 4 and p = 0.46 for Table 5).

## Discussion

This study examines the factors associated with walking to school and taking part in organised activities. Findings show that income is an important factor in determining the pattern of child activity levels at age 5. In general, walking to school is associated with lower income, while taking part in organised sports is associated with higher income. However, this finding is for children aged 5 and does not appear to be the same as that seen in older children, where more deprived children are reported to be less likely to walk [18,30]. However, this negative association for older children is not supported by all the literature [19]. For younger children (aged 5), it appears, with increasing income, the type of activity is exchanged from a free but time consuming activity (as most 5 year olds need to be accompanied to school if walking), to a time saving but fee paying activity. Interviews with older children (10-11 year olds) also suggest that physical activity for children in higher socio-economic schools is based around sports clubs, with parents providing financial support and transport to clubs, while, children from lower socioeconomic background have more unstructured play in the street and are verbally encouraged to "go out and do something" [31]. This swapping of activity may have implications when developing new interventions to increase physical activity in children. For example, interventions which encourage uptake of after school sports may have an effect of decreasing walking and overall activity levels may remain stable. Total activity for the individual would need to be considered in any evaluation of interventions aimed at improving any particular aspect of activity.

There are some families who appear to have high levels of general activity as measured by walking to school, playing organised sports and playing with the child. Activity levels were higher in families with evidence of good health behaviours (non-smoking, low BMI of mother, few hours of TV viewing) and those who were more affluent such as having a favourable socio-economic background or living in a 'good' area.

### Limitations of the study

However, how factors are associated with activity can not be fully deciphered from this study. For example, walking to school may be inversely associated with distance to school [10,32] rather than income. Distance to school was not measured in the cohort and is likely to be an important determinant of walking to school. In fact, increasing income may be associated with living in areas that are further away from the school of choice and, therefore, distance to school is likely to be an unmeasured confounder in this study. If distance to school was measured then the volume of active transport (distance and frequency) can also be calculated for each child. Importantly, this study also does not measure activities without the parent. There is no measure of unstructured play in the street (without the parents), and this may be an important source of activity in lower socio-economic children who are not attending clubs and sports. However, in children aged 5 this may have less importance than in older children. This study reports on the parent's opinion of their area which is subjective. There are no objective measurements of the built environment. In addition, this study asks parents how they take their child to school and if they take their child to organised activities. Parents self reported activity may be overestimated and there is no objective validation of the parents self reported activity with the child.

### Findings in context with other literature

This study does add to the debate regarding some previously published recommendations, suggesting priority should be given to lower socioeconomic status populations to facilitate environmental change and safety improvements to improve walking to school [33]. We did not find perceived safety of the area influenced walking (similar to Babey et al [34]). However, this could be because the parents were walking with the children. It is possible that at young ages safety was less of a consideration but it may be an important consideration for parents of older children [35]. Walking to school should be encouraged and targeted in "good" safe areas where working higher income parents live. For example, the Walking School Bus [36,37] may be most useful for the higher income working white families as organising a parent to walk with the child to school may be a barrier for families with both parents working. However, the design of the neighbourhood, distance to school, availability of pavements/sidewalks and ease of connectivity are all important considerations [38].

### Recommendations

In lower socio-economic areas, policies should also try to improve access to organised sports and clubs to encourage and facilitate involvement from ethnic minority and lower income families. It has been reported that participation in organised clubs/associations is an important part of children's activity, for example, in studies on teenagers this contributes 50-70% of their total physical activity [39,40]. However, participation would be improved if 'the cost was lower' and as cost has been reported to be a significant barrier to families with daughters and on lower income [21]. Thus, helping financially and improving physical access to sports and clubs could encourage parental support. Parental support is an important influence on the child's activity [41,42]. Education and playing with the child were found to be linked to both walking to school among higher income families and to involvement in organised sport among lower educational families. Thus, interventions targeted at improving general family health behaviours and support for physical activity appears to be beneficial across all communities. It has been shown [43] that having parents who value vigorous intensity sports provides the most benefits of activity and reduction of sedentary behaviour, while parents valuing household chores has unexpected negative effects (more TV viewing and decreased team participation). Attitudes vary by ethnicity, education, and number of children [43], all factors found to be associated with participation in organised sports/clubs within the MCS. Therefore, more research is needed to examine the family health beliefs and attitudes as predictors of the range of physical activities undertaken by children. Previous research [31] suggests that family based interventions need to accommodate the complex demands of two parent and single parent families and be affordable and varied enough to appeal to a wide range of interests and various ages and development stages of diverse ages of children in the same family.

### Conclusions

In summary, children from families that report adoptions of good health behaviours and engage in play with their children, show higher levels of physical activity. Thus, parenting practices appears to be an important part in the activity levels of children. This may include highlighting the importance of making time for playground visits and playing with your child. For example, there appears to be a time trade-off in some higher income families where parents either take the child to organised sports or play with their child but do not do both. Financially subsidising organised sports and exercise might help lower income families participate in more fee paying organised activities and this should be tested in a randomised controlled trial. However, this is likely to need to be combined with working with families to discuss attitudes and support for increased physical activity. Interventions aimed at one pattern of activity should be tested to ensure other forms of existing activity are not reduced.

## List of abbreviations

MCS: Millennium Cohort Study; BMI: Body Mass Index

## Competing interests

The authors declare that they have no competing interests.

## Authors' contributions

All authors were involved in designing the research question, SB and RC extracted and analysed the data supervised by MG, all authors read the first drafts of the manuscript, made amendments to the final document and approved the final manuscript.

## Pre-publication history

The pre-publication history for this paper can be accessed here:

http://www.biomedcentral.com/1471-2458/11/14/prepub
